# Rash and hepatosplenomegaly in a newborn

**DOI:** 10.1099/jmmcr.0.005098

**Published:** 2017-06-30

**Authors:** Eimear Kitt, Rebecca M. May, Andrew P. Steenhoff

**Affiliations:** ^1^​ Division of Infectious Diseases, Children’s Hospital of Philadelphia, Suite 1202 ARC 3615, Civic Center Boulevard, Philadelphia, PA 1910-4318, USA; ^2^​ Department of Pathology and Laboratory Medicine, University of Pennsylvania, 3400 Spruce Street, Philadelphia, PA 19104-4238, USA

**Keywords:** syphilis, TORCH infection, Spirochetes

## Abbreviations

CS, congenital syphilis; FTA-Abs, fluorescent treponemal antibody absorption ; IHC, immunohistochemical; RPR, rapid plasma reagin.

## Case summary

**Fig. 1.**
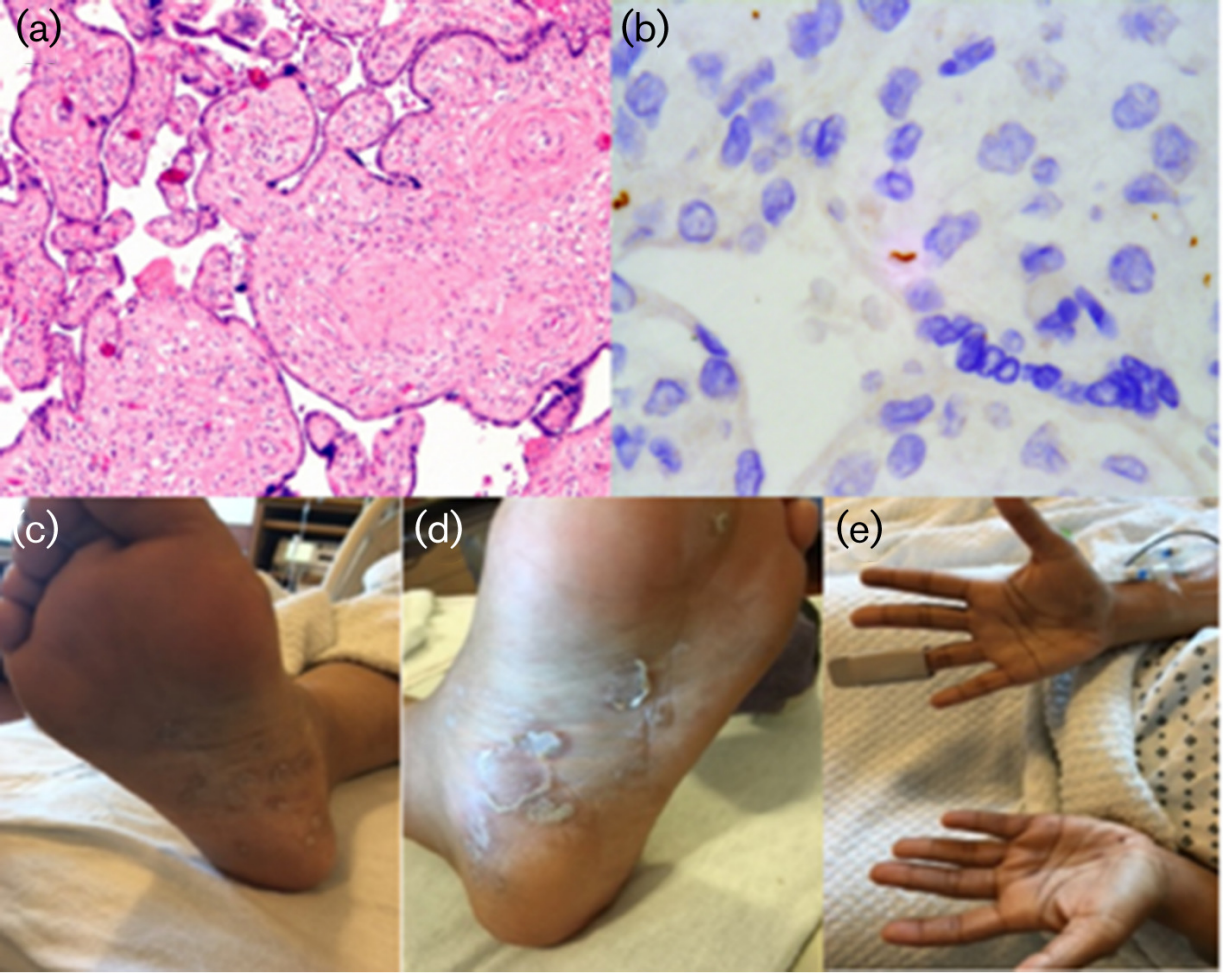
Pathology of the placenta from the infant with (a) 10× revaling perivillous fibrous proliferation or ‘onion skinning’ and (b) 100× revealing spirochete IHC stain, spirochetes are stained brown. (c–e) Maternal rash of secondary syphilis on the palms and soles of the hands and feet.

A newborn male infant, with an estimated gestational age of 35 weeks, was born via spontaneous vaginal delivery to a 28-year-old mother, who presented in active labour with a macular rash on her hands and feet noted at the time of delivery, as shown in Fig. 1(c–e). Maternal history was significant for illicit drug use and scant prenatal care, without thorough serological evaluation performed prior to delivery. Physical examination at birth revealed a small-for-gestational-age infant in moderate respiratory distress. He had a distended abdomen with hepatosplenomegaly. Dermatological examination demonstrated dry flaky skin throughout and some scattered petechiae. Laboratory evaluation was notable for haemoglobin of 13.3 g dl^−1^, leukocytosis of 81 000 µl^−1^ (55 % segmented neutrophils, 19 % monocytes, 21 % lymphocytes, 1 % bands and 2 % eosinophils) and thrombocytopenia of 15 000 µl^−1^ . The C-reactive protein level was markedly elevated at 47.8 mg l^−1^. Liver function tests unearthed a transaminitis and significant hyperbilirubinaemia at time of birth, which subsequently peaked at 16.6 µmol l^−1^ (direct 9.9, indirect 6.2). Radiographs of extremities revealed a ‘somewhat serrated appearance of proximal femoral and humeral metaphyses’. Brain imaging with head ultrasound was normal. Abdominal ultrasound confirmed enlargement of the liver. Treatment was started for a suspected congenital infection – confirmed based on the images of the placental tissue (Fig. 1a, b). He received two platelet transfusions, one red blood cell transfusion and was started on ursodiol for treatment of conjugated hyperbilirubinaemia, at a dose of 8 mg kg^−1^ divided twice daily. He responded to treatment and was well when discharged home at 2 weeks of life.

**Question**

Which of the following is the most likely diagnosis of the infant?

**Answer options**

1. Toxoplasmosis

2. Syphilis

3. CMV Cytomegalovirus

4. Listeria

## Discussion

**Correct Answer:** 2. Syphilis.

Maternal testing revealed rapid plasma reagin (RPR) positive status, confirmed with fluorescent treponemal antibody absorption (FTA-Abs), with a titre of 1 : 128. Further evaluation of the infant revealed he too was RPR positive with a titre of 1 : 16. His evaluation included a normal lumbar puncture with a nonreactive cerebrospinal fluid venereal disease research laboratory test. A diagnosis of congenital syphilis (CS) was made and the infant received a 10 day course of intravenous penicillin. G at a dose of 50 000 U kg^−1^ every 12 hours. At 2 and 3 months of age, FTA-Abs remained reactive, with titre decreased to 1 : 4 and 1 : 1, respectively. The infant was followed by gastroenterology staff for syphilis hepatitis with ongoing improvement. Notably, he had a normal neurodevelopmental outcome to date.

CS is a nationally notifiable disease that occurs when a mother transmits *Treponema pallidum* to her child. The reported rate of CS in the USA stood at 11.6/100 000 in 2014, which is the highest reported rate since 2001 [1]. CS is associated with a variety of significant obstetrical and neonatal complications, including miscarriage, stillbirth, preterm birth, neonatal death and neurodevelopmental disabilities [2, 3], with spirochetes able to cross the placenta from as early as 9–10 weeks gestation [4]. Our patient exhibited many common symptoms of early CS, including hepatomegaly (present in nearly all cases) [4] and splenomegaly (present in approximately 50 %) [3]. Jaundice is common and felt to be a result of either haemolytic anaemia or syphilis hepatitis. Haematological abnormalities including anaemia and thrombocytopenia are common, in addition to leukocytosis, as in this case. Mucocutaneous signs are well described, including the dry rash noted here. The ‘serrated appearance of proximal femoral and humeral metaphyses’ likely represents early manifestations of periostitis, pathognomonic to early CS [3].

The placental histological findings in CS are often non-specific. The umbilical cord often displays necrotizing funisitis, with necrosis and acute inflammation seen in the Wharton jelly around the umbilical vessels. The placental villi can show both acute and chronic villitis, and perivillous fibrous proliferation, termed ‘onion skinning’, may be prominent. If antibiotics have not yet been started at the time of delivery, a spirochete immunohistochemical (IHC) stain can be used to show organisms within the placenta and definitively make the diagnosis of CS. Notably, several of these features, including positive spirochete IHC staining, were seen in this case [5, 6].

The diagnosis of CS can be challenging for the health-care provider. A presumptive diagnosis is made using nontreponemal serological tests, such as the RPR test or venereal disease research laboratory test. Due to the possibility of a false-positive nontreponemal test, particular during pregnancy, a positive test must be confirmed by a specific treponemal test, such as the FTA-Abs test [7]. A definitive diagnosis of CS can be made with the visualization of spirochetes in infected tissue. This is particularly true if antibiotics have not yet been started at the time of delivery, when a spirochete IHC stain can be used to show organisms within the placenta [6] (Fig. 1).

The treatment of choice in both pregnancy and CS involves a parenteral penicillin regimen, due to documented efficacy in these settings [8, 9]. Even in the setting of drug allergy, there is no satisfactory alternative for the treatment of maternal syphilis, with desensitization recommended. For the mother, treatment duration depends on the diagnosed stage of syphilis. For the infant, the decision to treat depends on careful diagnostic evaluation, both from a clinical and serological perspective. As with all congenital infections, consultation with a paediatric infectious diseases physician is advised to optimize management. Close follow up of the child is also warranted to follow clinical progress, in addition to serial titres.

Despite the efforts of the World Health Organization to eliminate mother-to-child-transmission, syphilis poses an ongoing public-health threat to both maternal and foetal health. Our case highlights the typical findings unique to CS in the neonate, in addition to the rare but definitive diagnostic finding of positive spirochete staining in placental tissue. Health-care providers should be aware of the heightened prevalence of CS, to ensure prompt diagnosis and treatment, in the hopes of preventing the long-term sequelae of this easily treatable disease.
